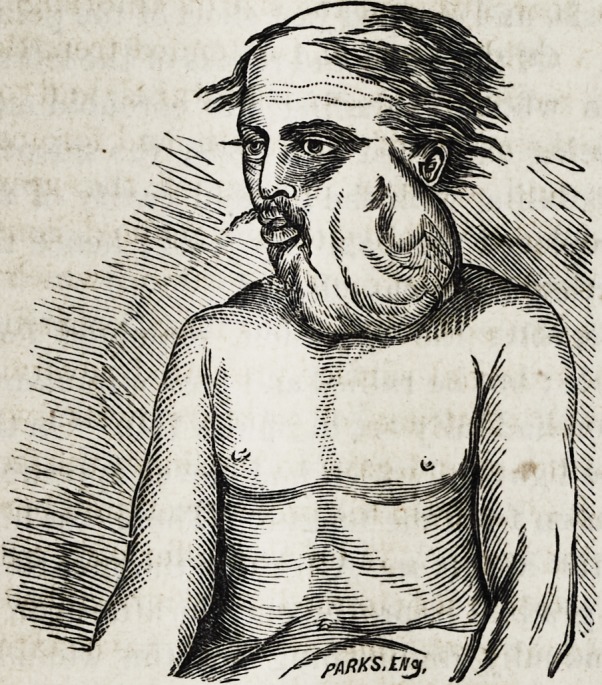# Large Multilocular Cystic Tumor of the Lower Jaw Removed by Excision

**Published:** 1869-10

**Authors:** W. B. Beatson

**Affiliations:** Civil Surgeon, Nagpore.


### SELECTED ARTICLES.

### ARTICLE VIII.

### *Large Multilocular Cystic Tumor of the Lower Jaw Removed by Excision.*

By W. B. Beatson, M.D., F.R.C.S., Civil Surgeon, Nagpore.

Pykajee, a Hindoo cultivator, aged about 35 years, made application at the Nagpore City Hospital in April, 1868, for relief on account of a tumor of the lower jaw, causing great pain and difficulty in mastication and deglutition. Three years previously he had first noticed a small swelling of the gum close to the molar teeth on the left side, which in the course of a year, attained the size of an orange. It was now as large as a child's head, and extended from the zygoma to the clavicle, carrying the ear backwards and outwards, almost filling the cavity of the mouth, and forcing the tongue, soft palate, and alveolar process of the upper jaw over towards the right. The mouth remained constantly open from the upward pressure of the tumor, which appeared on the mouth as an epitheliomatous mass, containing loosened teeth. The external surface presented several convexities, indicating the existence of separate cysts, was uniformly smooth and tense, and gave to the finger the feeling of crepitation which arises from the presence of fluid beneath thinned and distended bone. As he was suffering terrible pain from distension, a trocar was immediately introduced into the two most prominent cysts, one of which gave sixteen, the other twelve ounces of thick grumous and straw-colored fluids. No diminution took place in the size of the tumor, but the relief of tension was so great that pain almost entirely abated, and he was able to eat rice. Excision of the diseased bone was proposed to him and agreed to, but as he felt much better after the tapping, and as the proceeding had to be postponed for some days on account of the severe illness of the operator, the patient left the Hospital, promising to return when he had buried his mother-in-law, whose demise he declared had just occurred.

Nothing more was seen of him until the end of March, 1869, when he reappeared little altered in appearance. The tumour had, however, grown considerably; an extension of it was passing up under the zygoma, and commencing to distend the temporal fossa, and the cavity of the mouth was so much encroached upon that mastication was impossible, and deglutition very difficult. He was, of course, far from robust in condition; but as little was to be gained by delay, and the decease of his mother-in-law might be again expected at any moment in the event of his being frightened, the operation was performed with as little delay as possible on March 30, 1869.

A strong ligature was first passed through the point of the tongue, the first molar tooth on the right side was extracted, and the incision having been commenced, the bone was divided at that point with saw and nippers. The incision was extended to the articulation on the left, and the facial flap dissected upwards. Forcible depression now brought into view the soft portion, extending into the temporal fossa, and this being divided disarticulation was effected without any trouble, no real joint existing, and the tumor rapidly dissected from the submental portion of integument.

Lastly, the detached portion was enucleated from the temporal fossa. The bleeding was not great, and that from the facial vessels appeared to cause just sufficient faintness to check hæmorrhage from those divided in the later stage of the operation. Chloroform had been administered by Dr. John Law, ef the Madras Medical Service, and all vessels were promptly secured by my assistant, Baboo Gopal Chunder Roy.

The tumor weighed four pounds eleven ounces, and consisted of a number of cysts developed between the laminæ of the bone, mingled with an exuberant growth of epithelioma. The cystic dilatation of the bone extended beyond the symphysis, and stopped just short of the point of division by the saw. Of the body and ramus of the left side, no form was left, the whole, including the coronoid and condyloid processes, being expanded into cysts, the smooth surface of which, articulating with the glenoid cavity of the temporal bone, had replaced the maxillary joint; hence the ease with which disarticulation was effected.

The pulse, after the operation, remained for some time imperceptible; but he rallied and took stimulants. At four P. M. reaction had fully set in; pulse 150. On the following morning the ligature, which had never appeared necessary, was withdrawn from the tongue. There was high fever and a pulse of 150 at 4 P. M. After this the progress of the case was one of gradual improvement, the afternoon febrile exacerbation gradually diminishing and the wound healing by first intention, except at the point of exit of the ligatures.

On April 7 the ligatures had all separated. Speech and power of deglutition improved daily, and at the end of the month he left the Hospital to proceed to his home in satisfactory condition.—*London Med. Times & Gazette.*

## Figures and Tables

**Figure f1:**